# High Antimicrobial Resistance in ESKAPE Pathogens at a Rwandan Tertiary Hospital

**DOI:** 10.3390/pathogens14121253

**Published:** 2025-12-08

**Authors:** Charles Muhinda, Gad Murenzi, Leena Al-Hassan, Eric Seruyange, Leon Mutesa, Åsa Gylfe

**Affiliations:** 1 Department of Clinical Laboratory, Rwanda Military Referral and Teaching Hospital, Kigali P.O. Box 3377, Rwanda; muhindac@gmail.com (C.M.); ericseruyange@gmail.com (E.S.); 2 Center for Human Genetics and Genomics, College of Medicine and Health Sciences, University of Rwanda, Remera Campus-KG 11 Ave. 47, Kigali P.O. Box 4285, Rwanda; 3 Department of Research, Einstein Rwanda Research and Capacity Building Program, Kigali 00250, Rwanda; gadcollins@gmail.com; 4 Department of Global Health and Infection, Brighton and Sussex Medical School, Brighton BN1 9PS, UK; l.al-hassan@bsms.ac.uk; 5 Department of Clinical Microbiology and Umeå Centre for Microbial Research, Umeå University, SE90185 Umeå, Sweden

**Keywords:** antimicrobial resistance, ESKAPE pathogens, clinical isolates, referral hospital, Rwanda

## Abstract

Antimicrobial resistance (AMR) is a global health threat, increasing morbidity, mortality, and healthcare costs. Multi-drug resistant ESKAPE pathogens (*Enterococcus faecium*, *Staphylococcus aureus*, *Klebsiella pneumoniae*, *Acinetobacter baumannii*, *Pseudomonas aeruginosa*, and *Enterobacter cloacae*) cause most hospital-acquired infections. Local data on their resistance profiles remain limited in low-income settings. This study assessed the prevalence and resistance patterns of ESKAPE pathogens isolated from clinical specimens at Rwanda Military Referral and Teaching Hospital. A descriptive cross-sectional study was conducted from June 2022 to January 2023. ESKAPE isolates were identified and tested for antimicrobial susceptibility using the BD Phoenix M50 System. Data on sample type, ward, and demographics were analyzed. Of 744 bacterial findings, 207 (30%) were ESKAPE isolates. After excluding duplicates and non-recovered isolates, 156 were identified as ESKAPE. *K. pneumoniae* was most common (41%), followed by *S. aureus* (27%), *A. baumannii* (13%), *P. aeruginosa* (11%), and *E. cloacae* (8%); no *E. faecium* was detected. Among Gram-negatives, 63% were resistant to third-generation cephalosporins and 32% to carbapenems, with *A. baumannii* showing highest resistance (85% and 75%). Methicillin-Resistance in *Staphylococcus aureus* (MRSA) was 7%. This first hospital-based study in Rwanda shows high cephalosporin and carbapenem resistance, highlighting the need to strengthen diagnostics and stewardship.

## 1. Introduction

Antimicrobial resistance (AMR) presents a critical challenge to public health, particularly in low-income countries like Rwanda, where healthcare systems are already burdened with limited resources [1,2]. AMR is escalating morbidity, mortality, and healthcare costs, especially in regions with restricted access to diagnostic tools and non-functioning antimicrobial stewardship programs (AMS) [3]. Since the introduction of antibiotics in the 1940s, these drugs have been vital in revolutionizing medicine and improving health outcomes [4]. However, in many countries, the overuse and misuse of antibiotics have accelerated the rise in resistant bacterial infections, posing severe threats to healthcare services [5]. In Rwanda, studies have highlighted inappropriate prescribing practices, lack of access to diagnostic services for pathogen identification and susceptibility testing, and limited implementation of AMS programs [6]. Among the most worrisome pathogens are the ESKAPE group (*Enterococcus faecium*, *Staphylococcus aureus*, *Klebsiella pneumoniae*, *Acinetobacter baumannii*, *Pseudomonas aeruginosa*, and *Enterobacter cloacae*), which can acquire resistance to all available antibiotics [7]. *E. faecium* is commonly associated with urinary tract infections, bloodstream infections, and endocarditis, while *S. aureus* frequently causes skin and soft-tissue infections, surgical-site infections, pneumonia, and bacteremia [8,9]. *K. pneumoniae* often leads to hospital-acquired pneumonia, urinary tract infections, bloodstream infections, and neonatal sepsis [10]. *A. baumannii* and *P. aeruginosa* are important causes of ventilator-associated pneumonia, bloodstream infections, wound infections, and urinary tract infections [8,9,11]. *E. cloacae* causes urinary tract and device-associated infections [8]. These organisms are major causes of hospital-acquired infections (HAIs) and pose serious challenges for patient outcomes and healthcare systems, especially in countries like Rwanda, where the resources to combat AMR are limited [7]. The World Health Organization (WHO) has prioritized tackling ESKAPE pathogens, emphasizing the urgent need for new antibiotics and global surveillance systems [12]. However, comprehensive data on antimicrobial resistance patterns in Low and Middle-Income Countries (LMICs), including Rwanda, remain sparse [13]. This lack of data hinders effective monitoring, management, and the development of evidence-based guidelines for controlling AMR in these settings [13]. Research has consistently shown that infections caused by antibiotic-resistant bacteria lead to worse patient outcomes, including longer hospital stays, increased morbidity, and higher mortality rates. Treating AMR infections often requires more expensive and sometimes toxic antibiotics, further straining already overburdened healthcare systems, especially in LMICs [14]. In Rwanda, available studies, although limited, have reported high rates of extended-spectrum beta-lactamase (ESBL)-producing *K. pneumoniae* and *E. coli*, as well as increasing prevalence of methicillin-resistant *Staphylococcus aureus* (MRSA) [13,15,16]. There is a noticeable gap in research focusing on antibiotic resistance, particularly concerning ESKAPE pathogens in Rwanda. The absence of robust surveillance systems for AMR makes it challenging to fully understand the extent and dynamics of resistance patterns. Thus, context-specific studies are essential to inform local clinical practice and improve patient outcomes [17]. This study aims to evaluate the antimicrobial resistance patterns of ESKAPE pathogens isolated from clinical samples collected at Rwanda Military Referral and Teaching Hospital (RMRTH) between June 2022 and January 2023, providing data to inform locally relevant antibiotic stewardship, infection control, and treatment strategies in Rwanda.

## 2. Materials and Methods

### 2.1. Setting and Study Design

This cross-sectional study collected all putative *E. faecium*, *S. aureus*, *K. pneumoniae*, *A. baumannii*, *P. aeruginosa* and E. *cloacae* in routine bacterial culture at the microbiology unit at RMRTH, Kigali, between June 2022 and January 2023. The isolates were collected from different clinical samples including blood, urine, Cerebral Spinal Fluid (CSF), bronchial samples, and wounds. If more than one isolate was collected from a single patient, it was kept for the study only if it came from a different type of sample, was a different species, or showed a different antibiotic resistance pattern. RMRTH is a specialized healthcare facility offering advanced medical services and training in a diverse clinical environment. It receives both military and civilian patients and has a broad range of specialized wards including advanced surgery, adult and neonatal intensive care. For each stored isolate, information about the patient′s age, sex and type of sample was retrieved from the laboratory information system and stored in an Excel sheet. The total number of cultures with reported positive bacterial growth during the study period was collected from the laboratory log books and information system.

### 2.2. Bacterial Culture, Identification and Susceptibility Testing

All samples were processed as per the routine microbiological investigations protocol at RMRTH, using phenotypic and biochemical identification methods, including selective agar and analytical profile index 20 (API20E) (bioMérieux, Marcy-l’Etoile, France). Upon identification of the ESKAPE species, a loop full of the isolate was suspended in 5 mL sterile nutrient broth and incubated at 37° overnight. Next day, a 1 mL aliquot was transferred to a sterile plastic tube (2 mL) together with 100 µL 99% glycerol, and stored at −80° C until further use. The stored bacterial isolates were analyzed at the King Faisal Hospital, Microbiology laboratory, Kigali, Rwanda, to confirm bacterial species identification and antimicrobial susceptibility testing (AST), using BD Phoenix M50 Automated Microbiology System, Becton, Dickinson and Company (Franklin Lakes, NJ, USA). Prior to the analysis, samples were thawed at room temperature, and a full wire loop was inoculated in (sterile) nutrient broth and incubated at 37 °C overnight. The following day, a loop from the overnight culture was inoculated on agar plates (McConkey for Gram-negative and blood agar for Gram-positive) and incubated at 37 °C overnight. The BD Phoenix M50 Instrument (Becton, Dickinson and Company, Sparks, MD, USA) was used with BD Phoenix NMIC/ID-431 Panel (Becton, Dickinson and Company, Sparks, MD, USA) for identification and AST of Gram-negative bacteria and BD Phoenix™ PMIC/ID-111 Panel (Becton, Dickinson and Company, Sparks, MD, USA) for identification and AST of *Staphylococcus* species.

### 2.3. Data Analysis

Data were recorded in Excel sheet and analyzed using descriptive statistics to assess patient demographics, sample type, and referring department in relation to bacterial species and antibiotic resistance. AST was categorized as Sensitive, Intermediate, or Resistant (SIR) based on BD Phoenix outputs, which also identified ESBL, carbapenemase producers, and MRSA. For *K. pneumoniae* and *E. cloacae*, S categorization for ciprofloxacin, levofloxacin and cefazolin was not possible with the used kit, and non-resistant strains were therefore categorized “X”. The BD Phoenix SIR categorizations were consistent with Clinical Laboratory Standard Institute (CLSI) M100 ED35:2025 clinical minimum inhibition concentration (MIC) breakpoints [18].

### 2.4. Ethical Considerations

An application for full ethical approval was made to the Rwanda Military Hospital Institution Review Board and ethics consent was received on 21 June 2022. The ethics approval number is REF/92/RMH/COMDT/2022.s.

## 3. Results

### 3.1. Descriptive Data

#### 3.1.1. Isolate Characteristics

During the six-month study at RMRTH, 1994 cultures were performed, resulting in 744 positive cultures. From these, 207 ESKAPE isolates were cryo-stored, and 194 were successfully recovered and analyzed using the BD Phoenix system. After excluding 4 duplicate isolates with the same susceptibility patterns from the same patients and the same sample type (pus and tracheal aspirate), ESKAPE isolates from 132 patients were confirmed. Patient age and sex distribution are shown in Table 1.

The most common sample types were urine, pus and tracheal aspirate. There were only 21 blood culture isolates and 5 other sample types. Among the confirmed ESKAPE isolates, Gram-negative bacteria predominated, comprising 114 isolates (73.1%). *K. pneumoniae* was the most frequently isolated species, representing 64 (41.0%) of ESKAPE isolates, followed by *S. aureus* 42 (26.9%), *A. baumannii* 21 (13.5%), *P. aeruginosa* 17 (10.9%), and *E. cloacae* from 12 (7.7%) isolates (Table 2).

The 34 non-ESKAPE isolates were identified as other *Enterobacterales* (*n* = 14), other Gram-positive cocci (*n* = 6), Corynebacteria (*n* = 1), *Enterococcus* spp (*n* = 5), *E. faecalis* (*n* = 1), *A*. *lwoffii/haemolyticus* (*n* = 2), and other oxidase-positive environmental bacteria (*n* = 5). ESKAPE isolates were almost exclusively isolated from inpatients as only one isolate was sent from the polyclinic, managing the outpatients. The most common wards were internal medicine (IM) 44 (28.2%) and intensive care unit (ICU) 42 (26.9%) (Table 3).

#### 3.1.2. Antimicrobial Resistance Profiles

Full antimicrobial resistance profiles were achieved for most ESKAPE isolates although some isolates only had SIR categorization for a limited number of antibiotics. As shown in Table 4, Gram-negative isolates showed a low rate of susceptibility to cephalosporins. like ceftriaxone and ceftazidime (Table 4). Among 40 *K. pneumoniae* isolates resistant to ceftriaxone, 36 (90%) were identified as ESBL producers, and another three were positive for carbapenemase production. One isolate displayed resistance to cephalosporins and fluoroquinolones without defined mechanism.

In *E. cloacae*, only 3/11 isolates were susceptible to ceftriaxone, but no ESBL or carbapenemase was detected. Carbapenem susceptibility in *K. pneumoniae* and E. *cloacae* was high, with >90% of isolates susceptible to meropenem and imipenem, and >80% to ertapenem. In contrast, *P. aeruginosa* exhibited lower susceptibility rates to carbapenems, with 10/17 and 11/17 isolates susceptible to meropenem and imipenem respectively. Susceptibility to ceftazidime was observed in 9/17 isolates. Carbepenemase production was detected in one *P. aeruginosa* isolate.

Amikacin remained susceptible in almost all *K. pneumoniae*, *E. cloacae* and *P. aeruginosa* isolates. Susceptibility to ceftolozane–tazobactam was also relatively high (>75%) across these species. In *Acinetobacter baumannii*, resistance was widespread across β-lactams, aminoglycosides, and fluoroquinolones. However, 14 out of 20 isolates (70%) were susceptible to trimethoprim. Carbapenemase production was detected in 15 of the 20 *A. baumannii* isolates.

Susceptibility to fluoroquinolones could not be determined in *K. pneumoniae* and *E. cloacae*, but resistance to ciprofloxacin and levofloxacin was 24/63 (38%) and 10/63 (16%) respectively in *K. pneumoniae* and 7/11 (64%) and 0/11 (0%) respectively in E. cloacae. Among the *S. aureus* isolates, 3/42 were MRSA and resistant to oxacillin, 35/37 were susceptible to clindamycin and there was no resistance to vancomycin (Table 5).

## 4. Discussion

This study provides insights into the antibiotic susceptibility patterns of ESKAPE pathogens isolated from clinical specimens at a referral hospital in Rwanda, where ESKAPE comprised 30% of culture-positive isolates.

In this study, *K. pneumoniae* emerged as the most frequently isolated ESKAPE pathogen, accounting for 41% of all ESKAPE isolates. This predominance aligns with findings from local research in Rwanda, which highlight *K. pneumoniae* as a critical contributor to healthcare-associated infections [19]. For instance, a prospective cohort study at a Rwandan tertiary hospital identified *K. pneumoniae* among the leading pathogens responsible for surgical site infections (SSIs), emphasizing its impact on patient safety [19]. Our findings are also consistent with regional data, where *K. pneumoniae* remains a predominant and increasingly resistant pathogen. Studies from Ethiopia and Kenya have reported high rates of ESBL-producing *K. pneumoniae* with resistance to key antibiotics like ceftriaxone and amoxicillin/clavulanate [19,20]. In Rwanda, a 2021 to 2022 cohort study of internal medicine inpatients found *K. pneumoniae* and *E. coli* to be common pathogens, with only 27% of Gram-negative isolates susceptible to ceftriaxone [13]. Our study provides a broader perspective by including isolates from all hospital wards. Furthermore, a multicenter study (2020–2022) involving 1532 bloodstream infections in three tertiary hospitals also reported *K. pneumoniae* as the most common Gram-negative species. However, it showed lower susceptibility to cephalosporins (<15%) and carbapenems (<80%) compared to our findings [21]. These variations may be due to differences in study size, infection sites, patient populations, and quality of susceptibility testing and bacterial identification, underscoring the need for standardized AMR surveillance. In our study, cephalosporin resistance in *K. pneumoniae* was largely attributed to ESBL production. Carbapenem resistance was uncommon and observed even in isolates negative for carbapenemases, suggesting possible underdetection or involvement of other mechanisms such as AmpC beta-lactamases, porin loss, or efflux pumps. Due to laboratory limitations, further molecular confirmation was not performed, highlighting the urgent need to strengthen diagnostic capabilities in Rwanda. Although our method could not determine ciprofloxacin susceptibility for *K. pneumoniae* and *E. cloacae*, the observed high resistance rates (38% and 64%, respectively) suggest its empirical use should be limited and guided by AST results where possible. Globally, *K. pneumoniae* is recognized by the WHO as a critical-priority pathogen due to its resistance to last-line antibiotics, including carbapenems. According to the 2022 Global Antimicrobial Resistance Surveillance System (GLASS) report, it was among the top three most commonly reported resistant pathogens in hospital-acquired infections worldwide. Its capacity to form biofilms, persist in hospital environments, and transmit resistance plasmids makes it a formidable challenge for infection prevention and antimicrobial stewardship [22].

In this study, only 20 *A. baumannii* isolates were analyzed, of which 15 (75%) were resistant to carbapenems. Although intravenous colistin is often considered a last-resort treatment option for such infections [23], it was not tested because it was not included in the Gram-negative antibiotic panel used. Similar findings have been reported in Rwanda, where recent studies have identified *A. baumannii* as one of the most drug-resistant ESKAPE pathogens [6,13,21,24]. Data from tertiary hospitals in Kigali reported high levels of imipenem resistance among *Acinetobacter* spp [6,13,21,24]. This pattern is consistent with observations from neighboring and regional countries, confirming *A. baumannii* as a leading cause of carbapenem-resistant infections in healthcare settings across the region. Research conducted in Uganda reported carbapenem resistance rates as high as 87% among *A. baumannii* isolates from intensive care units, underscoring the pathogen’s critical role in healthcare-associated infections and its ability to withstand last-resort antibiotics [25]. Similarly, research from Tanzania and Kenya has documented the emergence of multidrug-resistant *A. baumannii*, often linked to prolonged hospitalization, limited infection prevention and control (IPC) measures, and the frequent use of broad-spectrum antibiotics [26,27]. These regional trends underscore the growing threat posed by *A. baumannii* in Sub-Saharan Africa, highlighting the urgent need for robust antimicrobial stewardship, improved diagnostic and IPC infrastructure challenges that mirror those observed in our study setting.

In this study, 29.4% of *P. aeruginosa* isolates were resistant to ceftazidime and 41.2% to meropenem, but only one was identified by the BD Phoenix system as a carbapenemase producer, suggesting other types of resistance mechanisms or possible under detection. These findings are consistent with reports from Tanzania [28], where meropenem resistance in *P. aeruginosa* is around 31%. Similar trends are observed in Uganda, with 45% resistance to ceftazidime, and even worse in Kenya [29], where ICU studies reported over 70% resistance to both drugs.

In this study, most *Staphylococcus aureus* isolates were oxacillin-susceptible, with only three MRSA cases and no vancomycin resistance. Clindamycin was effective against 95% of isolates. These results are consistent with findings from Rwandan tertiary hospitals prior to 2020, which documented a low prevalence of MRSA [13,16,30]. However, in the more recent study of blood cultures from three tertiary hospitals in Rwanda, oxacillin resistance varied and was up to 50% [21]. That may be due to either different epidemiology or lack of adequate identification methods and reagents in the published paper. No *E. faecium* was found, consistent with its rarity as a clinical cause of infection [31].

Our study is the first in Rwanda to comprehensively analyze AMR patterns of ESKAPE pathogens in a tertiary hospital setting. The use of both routine manual methods and subsequent confirmation with the BD Phoenix system provided a more reliable pathogen identification and susceptibility testing accuracy than in previous studies. In this study, 19.6% of isolates initially identified as ESKAPE pathogens using API 20E were later found to be different species when re-tested with the more accurate BD Phoenix system. This may be due not only to API limitations but also to errors during sub-culturing or storage, such as picking mixed or wrong colonies. Technical issues like labeling or handling errors may have further contributed. The automated BD Phoenix system also showed several limitations in this study. The BD Phoenix reagents available *in Rwanda* did not provide susceptible and intermediate results for fluoroquinolones in *K. pneumoniae* and *E. cloacae*. This limitation affects both the accuracy of resistance estimates and the ability to make informed treatment decisions, highlighting the need for supplementary testing. Another limitation of the study was the lack of documentation of the total number of ESKAPE isolates reported from the RMRTH lab during the study period, making it impossible to determine the dropout rate of isolates that were not stored for subsequent analysis. However, we estimate the dropout rate to be low. This is the first study in Rwanda to comprehensively assess automated resistance profiles of ESKAPE pathogens from a tertiary hospital. The BD Phoenix system improved diagnostic accuracy and highlights the need for quality-assured tools in LMICs to strengthen AMR surveillance. However, gaps in microbiology capacity and laboratory procedures may affect data reliability. This calls for confirmatory testing and stronger internal and external quality control measures.

As this manuscript provides an initial phenotypic overview, subsequent studies should focus on detailed genomic analyses, deciphering the genetic determinants of resistance and sequence types circulating in this part of the world.

The high prevalence of multidrug-resistant Gram-negative ESKAPE organisms identified in this study underscores the urgent need to strengthen IPC practices, AMS programs, and diagnostic capacity in Rwandan hospitals. These findings support the WHO Global Action Plan on AMR (2017) and highlight the critical need for sustained investments in laboratory infrastructure, workforce training, and integrated surveillance to mitigate the escalating threat of antimicrobial resistance in the region.

## 5. Conclusions

This study highlights the significant presence of ESKAPE pathogens in a Rwandan referral hospital setting, with *K. pneumoniae* being the most commonly recovered organism. The primary AMR challenges identified were resistance to third-generation cephalosporins among the majority of Gram-negative species, and carbapenem resistance notably among *A. baumannii* and *P. aeruginosa*. Most Gram-negative isolates remained susceptible to amikacin, with the exception of *A. baumannii*. MRSA was uncommon, and no *E. faecium* was detected. These findings underscore the importance of AMR surveillance in Rwanda to inform local antibiotic prescribing guidelines and strengthen infection prevention and control strategies. Additionally, a high degree of bacterial misidentification was observed, emphasizing the urgent need for quality control and the use of standardized, accurate identification methods.

## Figures and Tables

**Table 1:** Age and sex characteristics of study subjects.

Variable	Frequency	%
Sex		
Female	48	36.4
Male	84	63.6
Age		
Below 15	23	17.4
15–29	21	15.9
30–44	36	27.3
45–54	12	9.1
55 and above	40	30.3

**Table 2:** Distribution of ESKAPE isolates by clinical specimens.

Isolates	Blood Culture	Pus	Tracheal Aspirate	Urine	Others	Total
*A. baumannii*	1	2	15	3	0	21
*E. cloacae*	1	4	0	7	0	12
*K. pneumoniae*	7	9	13	34	1	64
*P. aeruginosa*	4	4	8	1	0	17
*S. aureus*	8	22	1	7	4	42
**Grand Total**	**21**	**41**	**37**	**52**	**5**	**156**

**Table 3:** Distribution of isolates by wards.

Wards	Isolates					
	*A. baumannii*	*E. cloacae*	*K. pneumoniae*	*P. aeruginosa*	*S. aureus*	Total
A&E	1	0	1	0	3	5
General surgery	0	3	4	0	3	10
Gynecology	0	2	10	0	1	13
ICU	16	0	16	10	0	42
Internal Medicine	3	3	16	5	17	44
Polyclinic	0	1	0	0	0	1
Neonatology	0	1	3	0	3	7
Pediatrics	0	1	5	2	15	23
Urology	1	1	9	0	0	11
**Grand Total**	**21**	**12**	**64**	**17**	**42**	**156**

Abbreviations: A&E: Accident and Emergency, ICU: Intensive Care Unit.

**Table 4:** Frequency of antibiotic susceptibility per Gram-negative ESKAPE pathogens.

Antibiotics	*A. baumannii*	*E. cloacae*	*K. pneumoniae*	*P. aeruginosa*
Amikacin	6/20 (20.0%)	10/10 (100.0%)	62/63 (98.4%)	17/17 (100.0%)
Gentamicin	3/20 (15.0%)	5/11 (45.4%)	45/63 (71.4%)	13/17 (76.4%)
Amoxicillin–Clavulanic acid	NA	0/12 (0.0%) ^a^	34/63 (53.9%)	NA
Ampicilin	NA	0/12 (0.0%) ^a^	0/63 (0.0%)	NA
Cefazolin	NA	0/12 (0.0%) ^a^	0/63 (0.0%)	NA
Cefuroxime	NA	2/11 (18.1%)	21/63 (33.3%)	NA
Ceftazidime	3/18 (16.6%)	NA	NA	9/17 (47.3%)
Ceftriaxone	3/20 (15.0%)	3/11 (27.2%)	23/63 (36.5%)	NA
Cefepime	4/20 (20.0%)	2/11 (18.2%)	25/63 (39.6%)	9/17 (52.9%)
Ceftolozane–Tazobactam	NA	9/11 (81.8%)	51/62 (82.2)	13/17 (76.4%)
Piperacillin–Tazobactam	2/20 (10.0%)	7/11 (63.6%)	47/63 (75%)	9/17 (52.9%)
Ertapenem	NA	9/11 (81.8%)	55/63 (87.3%)	NA
Imipenem	5/20 (25.0%)	11/11 (100.0%)	61/63 (96.8%)	11/17 (64.7%)
Meropenem	5/20 (25.0%)	10/11 (90.9%)	59/63 (93.6%)	10/17 (58.8%)
Tigecycline	NA	7/8 (87.5)	46/52 (88.4%)	NA
Ciprofloxacin	4/20 (20.0%)	0/11 (0.0%) ^a^	0/63 (0.0%) ^a^	11/17 (64.7%)
Levofloxacin	4/20 (20.0%)	NA	0/63 (0.0%) ^a^	11/17 (64.7%)

^a^ The Result S was not possible to obtain with the phoenix cartridge used in the study. NA = not analyzed.

**Table 5:** Antibiotic susceptibility for Staphylococcus aureus.

Antibiotics	Frequency of Antibiotic Susceptibility
Trimethoprim–Sulfamethoxazole	16/39 (41.0%)
Nitrofurantoin	5/6 (83.3%)
Daptomycin	42/42 (100.0%)
Linezolid	41/41 (100.0%)
Oxacillin	39/42(92.8%)
Penicillin G	0/41 (0.0%)
Rifampin	41/42 (97.6%)
Tetracycline	32/41 (78.0%)
Vancomycin	42/42 (100.0%)
Erythromycin	35/37 (94.5%)
Clindamycin	35/37 (94.5%)
Ceftaroline	42/42 (100.0%)

## Data Availability

The raw data supporting the conclusions of this article will be made available by the authors upon request.

## Abbreviations

The following abbreviations are used in this manuscript:

AMR	Antimicrobial Resistance
AMS	Antimicrobial Stewardship
API20Es	Analytical Profile index 20 Enterobacterales
BD Phoenix™	Becton, Dickinson Phoenix™ system
CLSI	Clinical Laboratory Standard Institute
CSF	Cerebral Spinal Fluid
ESBL	Extended-Spectrum Beta-Lactamase
ESKAPE	*Enterococcus faecium*, *Staphylococcus aureus*, *Klebsiella pneumoniae*, *Acinetobactor baumannii*, *Pseudomonas aeruginosa*, *Entrobacter cloacae*
GLASS	Global Antimicrobial Surveillance System
HAI	Hospital Acquired Infection
ICU	Intensive Care Unit
IPC	Infection Prevention and Control
LMICs	Low and Middle-Income Countries
MDRO	Multi-Drug Resistant Organism
MIC	Minimum Inhibition Concentration
MRSA	Methicillin-Resistant Staphylococcus Aureus
MRSA	Methicillin-Resistant *Staphylococcus aureus* (MRSA)
NMIC/ID	Negative Minimum Inhibition Concentration Identification
PMIC/ID	Positive Minimum Inhibition Concentration Identification
RMRTH	Rwanda Military Referral and Teaching Hospital
SIR	Sensitive, Intermediate, Resistant
SSIs	Surgical Site Infections
WHO	World Health Organization
